# Pulmonary mucormycosis in a patient with uncontrolled diabetes

**DOI:** 10.1590/0037-8682-0168-2024

**Published:** 2024-06-10

**Authors:** Luciana Volpon Soares Souza, Arthur Soares Souza, Gláucia Zanetti, Edson Marchiori

**Affiliations:** 1 Ultra X, São José do Rio Preto,SP, Brasil.; 2 Faculdade de Medicina de São José do Rio Preto, SP, Brasil.; 3 Universidade Federal do Rio de Janeiro, Rio de Janeiro, RJ, Brasil.

A 54-year-old obese woman with uncontrolled diabetes mellitus (glycemia, 644 mg/dL; glycated hemoglobin, 21.7%) presented with a 2-week history of fever and cough. The serological test results for human immunodeficiency virus infection were negative.

**FIGURE 1:**
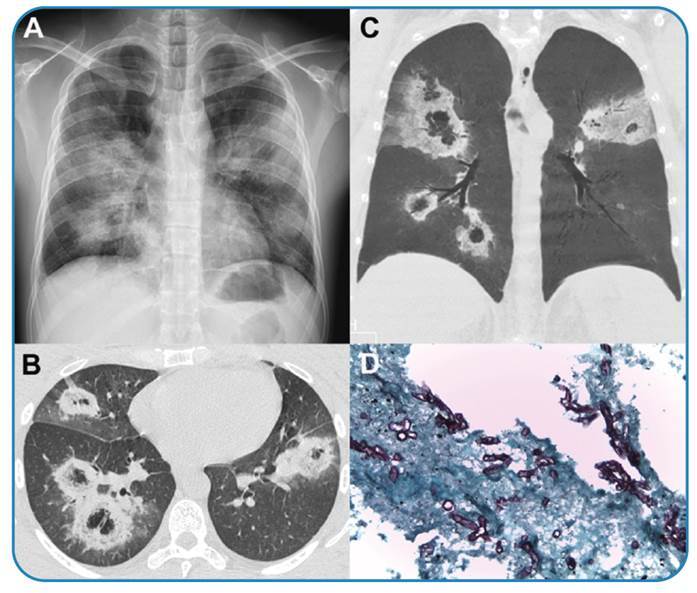
**(A)** Chest radiograph showing rounded focal consolidations with hypertransparent centers in both lungs. Chest computed tomography images with axial **(B)** and coronal reconstruction with minimum intensity projection **(C)**, demonstrating multiple reversed halo signs in both lungs, with low-attenuation areas inside the halos and thick outer rims of consolidation. **(D)** Grocott’s special stain identifying the presence of fungal hyphae, confirming the diagnosis of mucormycosis (×100).

Chest radiography performed on admission showed nonhomogeneous consolidations in both lungs (Figure 1A). Chest computed tomography revealed multiple reversed halo signs (RHSs) in both lungs, with low attenuation areas inside the halos and thick outer rims of consolidation (Figure 1B, C ). A pulmonary biopsy revealed yeast and hyphae suggestive of mucormycosis (Figure 1D). Therefore, the patient was diagnosed with pulmonary mucormycosis. The patient had a poor prognosis and died 8 days after admission.

Pulmonary mucormycosis, previously known as zygomycosis, is a severe, invasive lung infection caused by filamentous fungi belonging to the order Mucorales. It occurs almost exclusively in diabetic and immunocompromised patients and has a mortality rate exceeding 50%^1^ ^,^ ^2^.

The clinical signs and imaging findings of pulmonary mucormycosis are nonspecific, although the presence of RHS in patients with neutropenia is highly suggestive of the disease. Some morphological characteristics of RHS contribute to diagnostic suspicion. Reticulation inside an RHS with an outer consolidation rim > 1 cm thick, strongly suggests invasive fungal infections, particularly pulmonary mucormycosis^3^ ^-^ ^5^. Although the definitive diagnosis should be based on a biopsy, with the identification of hyphae in infected tissues, the presence of an RHS with these morphological characteristics should be sufficient for the early initiation of appropriate therapy, thereby improving the outcome.
